# Association Between Metabolic Syndrome and Decline in Cognitive Function: A Cross-Sectional Study

**DOI:** 10.2147/DMSO.S393282

**Published:** 2023-03-21

**Authors:** Hissa N Alsuwaidi, Ashraf I Ahmed, Hamad A Alkorbi, Sara M Ali, Lina N Altarawneh, Shooq I Uddin, Sara R Roueentan, Asmaa A Alhitmi, Laiche Djouhri, Tawanda Chivese

**Affiliations:** 1 College of Medicine, QU Health, Qatar University, Doha, Qatar

**Keywords:** metabolic syndrome, cognitive dysfunction, Middle East and North Africa

## Abstract

**Aim:**

We investigated whether metabolic syndrome (MetS) is associated with a decline in cognitive function in a cohort of middle-aged and elderly individuals without known cognitive dysfunction diseases in Qatar.

**Methods:**

We conducted a cross-sectional study on randomly selected participants aged 40–80 years from the Qatar Biobank, with data on cognitive tests and MetS components. Participants with a history of dementia, stroke, or mental disorders were excluded. MetS was diagnosed using the NCEP-ATP III criteria and cognitive performance was assessed using the Cambridge Neuropsychological Test Automated Battery (CANTAB). Two cognitive function domains were assessed. These are speed of reaction, measured using the Reaction Time (RT), and short-term visual memory, measured using the Paired Associate Learning (PAL) test. Multivariable logistic regression models were used to determine associations between MetS and poor speed of reaction and poor memory performance.

**Results:**

The mean age of the participants included was 49.8 years (SD 6.7). Of these, 51.9% were females and 88.0% were of Qatari nationality. Most of the 1000 participants had MetS (n=302) or 1–2 MetS components (n=523), whereas only 170 had no MetS components. There was a strong association between MetS and poor memory performance (OR 1.76, 95% CI 1.04–2.96, P=0.034), but a weaker association with poor speed of reaction (OR 1.5, 95% CI 0.89–2.50, P=0.125).

**Conclusion:**

In middle-aged and elderly individuals, MetS was strongly associated with diminished short-term visual memory, psychomotor coordination and motor speed.

## Introduction

Metabolic syndrome (MetS) is a cluster of metabolic risk factors known to increase the risk of cardiovascular disease and death, and is a global problem that affects nearly 20–25% of adults^1^ and about 28% of Qatari adults.^2^ The risk factors, which are the components of MetS, include raised blood pressure, visceral obesity, dysglycemia and dyslipidaemia. Although MetS is mostly known as a risk factor for cardiovascular disease,^3^ there is some evidence that it may lead to worsening cognitive function, especially in older adults. However, the association between MetS and cognitive dysfunction remains debatable.^6^

It may not be possible to restore cognitive function if it declines, especially in older adults.^4^ Therefore, identifying individuals who are susceptible to cognitive dysfunction would be of great value to reduce the associated decrease in quality of life. One prospective cohort study of Danish postmenopausal women showed that impaired fasting plasma glucose was the one metabolic risk factor that showed a strong association with cognitive dysfunction.^5^ However, results from other longitudinal studies were inconclusive, perhaps due to differences in age, gender, genetic predisposition, length of follow-up, and type of cognitive test. Although one meta-analysis showed that MetS increases the risk of mild cognitive impairment,^6^ the findings were not conclusive, and this area remains under-researched, therefore, warrants further investigations.

Several important knowledge gaps remain concerning MetS and cognitive function. First, data on the association between cognitive dysfunction and MetS are scarce in middle-aged individuals.^7^ In addition, the association between MetS and cognitive dysfunction remains debatable. Indeed, some studies have reported that MetS is an independent risk factor for cognitive impairment, whereas other studies found no significant association between MetS and cognitive function. Further, findings on associations between MetS and domain-specific cognitive function remain limited.^6^ Lastly, data on the association between MetS and cognitive function is lacking in the Middle East and North Africa regions, which are regions with a high prevalence of MetS. Therefore, the aim of this current study was to contribute to this relatively under-researched field in the Middle East by investigating the association between MetS and decline in cognitive function in middle-aged and elderly participants without any cognitive disorders in Qatar. Additionally, we investigated the association between the individual components of MetS and decline in cognitive function.

## Material and Methods

### Study Design and Setting

We conducted a cross-sectional study that included participants who were randomly chosen from the Qatar Biobank (QBB), which is a nationwide longitudinal cohort and repository of samples and data on different aspects of health and lifestyle of participants, who are citizens and long-term residents of Qatar.^8^ The design and methods of the QBB have been described previously.^8^ In the current study, we will refer to the methods used by the parent QBB. The data that were obtained from QBB included data on the Cambridge Neuropsychological Test Automated Battery (CANTAB), demographics, laboratory results, and comorbidities. Men and women aged 40–80 years who had cognitive test results were included in this study. Participants with a history of dementia, stroke, or mental disorders were excluded.

### Collected Data

The following data were obtained for each participant from the QBB: demographic data, such as age, gender, education level, marital status, nationality, occupation, and family medical history. Data on chronic disease history, blood pressure and anthropometric measurements (BMI, waist-to-hip ratio, systolic blood pressure, diastolic blood pressure, mean arterial pressure, and use of antihypertensive medication), history of cardiovascular diseases (confirmed diagnosis of stroke and acute coronary syndrome), data on diabetes (confirmed diagnosis of diabetes, fasting glucose levels, glycated haemoglobin, and use of diabetic medications), and data on dyslipidaemia (levels of HDL, LDL, cholesterol, triglycerides, and use of lipid-lowering agents). Further, data on smoking history (cigarette, shisha, passive smoking), diet, physical activity, and cognitive test data were obtained.

### Metabolic Syndrome (MetS)

MetS was diagnosed using NCEP-ATP III criteria.^9^ For participants to be considered to have MetS; they must have three or more of the following risk factors: dysglycaemia (HbA1C ≥ 5.7%) or previously diagnosed with type 2 diabetes mellitus, raised blood pressure (systolic ≥ 130 mmHg and/or diastolic ≥ 85 mm Hg), dyslipidaemia which is defined as triglycerides ≥1.7 mmol/L or low HDL-C (men – less than or equal 1.03 mmol/l, women – less than or equal 1.3 mmol/l), and abdominal obesity (waist circumference ≥ 102 cm in men, waist circumference ≥ 88 cm in women). Participants in this study were divided into three groups; those with MetS (at least 3 MetS components), those with 1–2 MetS components but not meeting the strict definition of MetS and those without any MetS components.

### Assessment of Cognitive Function

The CANTAB (Cambridge Neuropsychological Test Automated Battery) system was used to measure cognitive function. Two tests were used to assess cognitive function at the Qatar Biobank, which are the Reaction Time (RT) test that measures psychomotor coordination and motor speed, and the Paired Associate Learning (PAL) test that measures short-term visual memory.^10^

The RT consists of 60 trials per participant, where the participant must keep their finger on a button that is presented at the bottom of the screen. The participants must then react as fast as they can to select the target that will be presented as a small box within one of the two larger black boxes. This test evaluates the time needed to respond and the total mistakes a participant made in those 60 trials. We generated a novel reaction score, which was the product of the total mistakes a participant made in the 60 trials multiplied by the time they took. The generated score reflects both variables collectively, and therefore the participant with the lowest score had the best performance.

On the other hand, the PAL test evaluates the visual short-term memory function of participants via displaying a series of images located within certain boxes on the screen in which participants must memorize them within a short time period. The images displayed in the boxes were then hidden. After that, the participant would see a certain image in the middle of the screen where they are expected to match this image to the box that displayed it. The test involves a total of 7 levels, and the participant moves to a higher level of difficulty with more boxes if all the shapes were matched correctly. However, a total of 10 unsuccessful attempts at any level would terminate the test. This test reports the number of attempts needed to correctly identify the location of each target at any level, the maximum level of difficulty reached by the participant, and the total time needed to end the test. Based on the test results, we generated a novel memory scoring scheme that incorporates all the aforementioned variables to rank the participants from 1 to N by sorting on levels of difficulty, total mistakes, and time taken (in that order), with difficulty level given the highest priority and time taken given the least priority. The generated score reflected all variables, as shown in the example in Supplementary Table 1. The numerical ranking was then converted into a percentile and those below the 25^th^ percentile were considered to have significantly lower cognitive function, compared to the rest of the sample. The participants were then classified into poor memory performance (below 25th percentile) and normal, and this served as the binary variable for our analysis.

### Statistical Analysis

First, participants were divided into two groups, one with MetS and the other without MetS, according to NCEP-ATP III criteria. Then, we subdivided the latter group into those with 1–2 MetS components and those who were totally free of the MetScomponents of interest. The participants’ demographics and characteristics as well as their performance scores in both tests (RT and PAL) were compared between the three groups. For categorical variables, frequencies and proportions were used to summarise the data. The Chi-squared test and Fisher's Exact (when there were small frequencies) were used to compare categorical variables. The continuous data were first tested for normality using histograms. Continuous variables were described as mean and standard deviations (SD) if normally distributed or as median and interquartile ranges if the variables were not normally distributed. ANOVA was used to compare continuous variables across the MetS groups if data were normally distributed. For the variables that were not normally distributed, comparisons were tested using the Kruskal–Wallis test. Post hoc tests were done with the Bonferroni correction for the comparison groups only when ANOVA P≤0.05.

We compared cognitive function by MetS status using the generated scores for both tests (RT & PAL). For the primary analysis, the presence of MetS was defined as the independent variable to predict the changes in the performance scores in either of the tests (dependent variables). Both continuous outcome scores did not meet the assumptions of linear regression, as their residuals were skewed. Thus, we identified cut-off values for RT and PAL scores using the quartiles to categorize the poorest performers in the two cognitive tests. For the PAL test, a cut-off value below the 25^th^ percentile was indicative of poor memory performance in comparison to all participants. A score value above the 75^th^ percentile reflected poor reaction performance for the RT test. The cut-offs were then used to categorize the continuous performance scores, into binary outcomes. We used logistic regression to investigate the association of metabolic syndrome with a poor performance score in each test independently. The components of metabolic syndrome such as (dysglycemia, dyslipidaemia, elevated blood pressure, and abdominal obesity) were also evaluated for associations with poor performance in either the RT or PAL test. Odds ratios (ORs), their 95% CIs, and P-values were reported. We adjusted for confounders identified through a directed acyclic graph (DAG), namely; age, sex, income, physical activity, diet, cigarette smoking, education, and shisha smoking.

### Ethics

Ethics approval for this study was provided by the Qatar Biobank (QBB) (Ref - EX-2019-RES-ACC-0182-0107). During their participation in the QBB, participants gave written informed consent. The research also received waiver of ethics approval from the Qatar University Institutional Review Board (Ref - QU-IRB 1223-E/20).

## Results

### Characteristics of Participants by Metabolic Syndrome

Of the 1000 participants included in the present study, 175 were with no MetS components, 523 with 1–2 MetS components, and 302 with MetS. The characteristics of the participants are shown in Table 1. Overall, the mean age was 49.8 years (SD 6.7 years, range 41–69 years), 51.9% were females and 88.0% were of Qatari nationality. Post hoc analysis showed that the MetS group had a higher median age, increased BMI, raised waist circumference, elevated HbA1c, raised triglycerides, low HDL, and raised compared to the other two groups, with strong evidence against the null hypothesis in all these comparisons (Supplementary Table 2).

**Table 1:** Characteristics of Participants by Metabolic Syndrome

Factor	No MetS (No Components)	No MetS (1–2 Components)	MetS	*P*-value
**N**	175	523	302	
**Age, median (IQR)**	46.0 (43.0, 49.0)	48.0 (44.0, 54.0)	50.0 (46.0, 56.0)	<0.001
**BMI, mean (SD)**	27.0 (3.7)	29.6 (5.2)	32.3 (5.3)	<0.001
**Gender**				
** Female**	100 (57.1%)	263 (50.3%)	156 (51.7%)	
** Male**	75 (42.9%)	260 (49.7%)	146 (48.3%)	0.29
**Nationality**				
** Non-Qatari**	17 (9.7%)	58 (11.1%)	45 (14.9%)	
** Qatari**	158 (90.3%)	465 (88.9%)	257 (85.1%)	0.16
**Waist Circumference**				
** Normal**	175 (100.0%)	346 (66.2%)	82 (27.2%)	
** Raised**	0 (0.0%)	177 (33.8%)	220 (72.8%)	<0.001
**HbA1c**				
** Normal**	175 (100.0%)	250 (47.8%)	47 (15.6%)	
** Elevated**	0 (0.0%)	273 (52.2%)	255 (84.4%)	<0.001
**Triglycerides**				
** Normal**	175 (100.0%)	411 (78.6%)	99 (32.8%)	
** Raised**	0 (0.0%)	112 (21.4%)	203 (67.2%)	<0.001
**HDL**				
** Normal**	175 (100.0%)	409 (78.2%)	98 (32.5%)	
** Decreased**	0 (0.0%)	114 (21.8%)	204 (67.5%)	<0.001
**Blood pressure**				
** Normal**	175 (100.0%)	423 (80.9%)	150 (49.7%)	
** Raised**	0 (0.0%)	100 (19.1%)	152 (50.3%)	<0.001
**Diet**				
** Less than 3 times/ week**	167 (97.1%)	497 (96.7%)	290 (97.0%)	
** More than 3 times/ week**	5 (2.9%)	17 (3.3%)	9 (3.0%)	0.95
**Physical activity**				
** Yes**	48 (27.4%)	143 (27.3%)	63 (20.9%)	
** No**	127 (72.6%)	380 (72.7%)	239 (79.1%)	0.095
**Education**				
**Below tertiary education**	48 (27.4%)	197 (38.0%)	129 (42.9%)	
**Tertiary education and above**	127 (72.6%)	322 (62.0%)	172 (57.1%)	0.004
**Cigarette smoking**				
** Smokers**	24 (21.6%)	66 (20.6%)	42 (23.1%)	0.63
** Ex-smokers**	12 (10.8%)	38 (11.8%)	28 (15.4%)	
** Non-smokers**	75 (67.6%)	217 (67.6%)	112 (61.5%)	
**Shisha smoking**				
** Yes**	41 (36.3%)	117 (36.0%)	57 (31.3%)	0.53
** No**	72 (63.7%)	208 (64.0%)	125 (68.7%)	

### Comparison of Memory Performance by MetS Status

**Figure 1:**
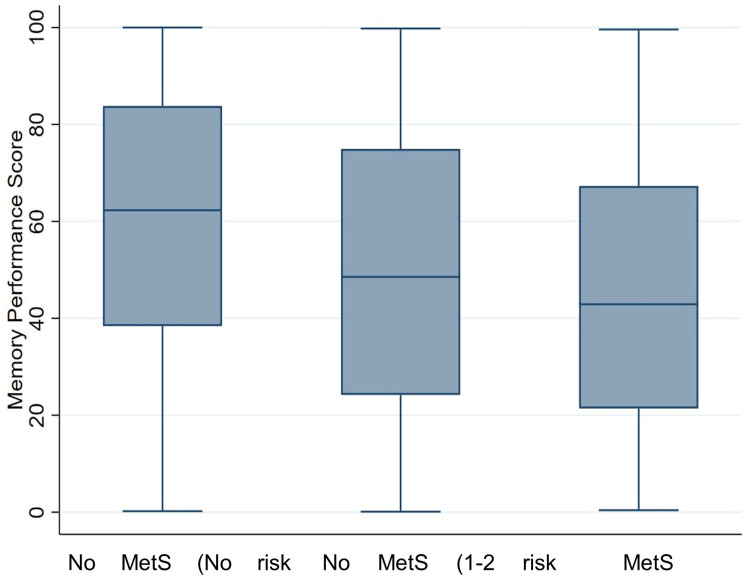
Box plot showing comparison of memory performance score by MetS status. NB: The memory score is a ranking of participants based on highest difficulty level reached, total mistakes, and the total time taken to complete the test. Participants who reached to the maximum level of difficulty with lowest mistakes and shortest duration of time were assigned a higher ranking in comparison to the other participants and the participant with the lowest rank had the worst memory performance score.

Table 2 and Figure 1 show comparisons of memory performance by MetS status. The group with MetS had worse memory test perfomance, with a lower median memory performance score compared to the group with no MetS (median 42.9, IQR 21.4–67.3, median 62.3, IQR 38.4–83.8; respectively), with very strong evidence against the null hypothesis (P<0.001). Further, the group with MetS had a lower memory performance median compared to the group with MetS 1–2 components (median 42.9, IQR 21.4–67.3, median 48.6, IQR 24.2–74.9; respectively), with little evidence against the null hypothesis at this sample size (P=0.117). In addition, the group with 1–2 MetS components also had a lower memory performance median compared to the group with no MetS (median 48.6, IQR 24.2–74.9, median 62.3, IQR 38.4–83.8; respectively), with strong evidence against the null hypothesis (P=0.003).

**Table 2:** Comparison of Memory Performance and Speed of Reaction Score by Metabolic Syndrome

Factor	No MetS (No Components)	No MetS (1–2 Components)	With MetS	*P*-value
N	175	523	302	
Memory performance, median (IQR)	62.3 (38.4, 83.8)	48.6 (24.2, 74.9)	42.9 (21.4, 67.3)	P<0.001*
Speed of reaction score, median (IQR)	82.5 (49.2, 168.3)	87.7 (51.8, 168.8)	105.7 (55.9, 219.2)	P*<* 0.01**

**Notes**: Post hoc analysis (using Bonferroni adjustment): *MetS vs No MetS (1–2 components), P= 0.117. MetS vs No MetS (no components), P< 0.001. No MetS (1–2 components) vs No MetS (no components), P=0.003. **MetS vs No MetS (1–2 components), P= 0.012. MetS vs No MetS (no components), P=0.007. No MetS (1–2 components) vs No MetS (no components), P=0.586.

For MetS components, participants with dysglycaemia, raised blood pressure, and raised waist circumference performed worse, with lower median scores in memory performance, compared to those without those components (Supplementary Table 3, and Supplementary Figure 1). However, there were no differences in memory performance scores between participants with dyslipidaemia (low HDL, raised triglycerides), and those without dyslipidaemia (Supplementary Table 3, and Supplementary Figure 1).

### Comparison of Speed of Reaction by MetS Status

**Figure 2:**
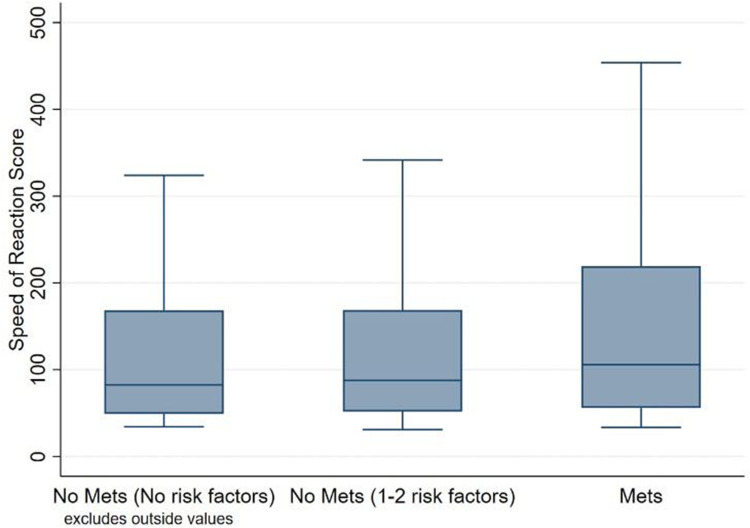
Box plot showing comparison of speed of reaction performance by MetS status. NB: The reaction score is a product of the total mistakes a participant made in 60 trials multiplied by the time they took. Therefore, the participant with the lowest score had the best performance.

Table 2 and Figure 2 show comparisons of speed of reaction by MetS status. The group with MetS had worse speed of reaction, with a higher speed of reaction score median compared to the group with no MetS (median 105.7, IQR 55.9–219.2, median 82.5, IQR 49.2 −168.3; respectively), with strong evidence against the null hypothesis (P=0.007). Further, the group with MetS had worse speed of reaction, with a higher speed of reaction score median compared to the group with 1–2 MetS components (median 105.7, IQR 55.9–219.2, median 87.7, IQR 51.8 −168.8; respectively), with moderate evidence against the null hypothesis (P=0.012). However, there were no clinically and statistically significant differences in the speed of reaction score between the individuals with no MetS and those with 1–2 MetS components (Table 2 and Figure 2).

For MetS components, participants with dysglycaemia and raised waist circumference had a worse speed of reaction performance with higher median scores compared to those without those components (Supplementary Table 3, and Supplementary Figure 2). However, there were no differences in speed of reaction scores between participants with dyslipidaemia (low HDL, raised triglycerides), and raised blood pressure compared to those without the components of interest (Supplementary Table 3, and Supplementary Figure 2).

### Association Between MetS and Cognitive Dysfunction – Multiple Variable Logistic Regression

#### Memory Performance

After multivariable logistic regression, MetS was associated with clinically significant higher odds of poor memory performance compared to having no MetS (OR 1.76, 95% CI 1.04 −2.96), with moderate evidence against the null hypothesis (P=0.034). Having 1–2 MetS components showed a 62% increase in the odds of poor memory perfomance (OR 1.62, 95% CI 1.00–2.66), but with weak evidence against the null hypothesis (P=0.051) (Table 3). Furthermore, all components of MetS, except triglycerides, showed an increase in odds of poor memory performance, albeit, with little evidence against the null hypothesis (Supplementary Table 4). Upon stratification by education level, compared to those without MetS, participants with MetS who did not attain tertiary education showed a 2.5-fold increase in the odds of having poor memory performance score (OR 2.51, 95% CI 1.08 −5.83), with moderate evidence against the null hypothesis (P=0.032), while weaker associations were observed in those with tertiary education (Supplementary Table 5).

**Table 3:** Association Between Metabolic Syndrome and Cognitive Dysfunction – Multivariable Logistic Regression

Unadjusted Analysis				Adjusted Analysis		
	OR	95% CI	*P*-value	OR	95% CI	*P*-value
**Memory performance score**						
No MetS components	Ref					
1–2 MetS components	2.08	1.31 −3.32	0.002	1.62	1.00 −2.66	0.051
MetS	2.56	1.57 −4.18	0.001	1.76	1.04 −2.96	0.034
Constant (baseline odds)	0.17			0.89		
**Speed of reaction score**						
No MetS components	Ref					
1–2 MetS components	1.20	0.76 −1.89	0.442	1.11	0.64–1.80	0.677
MetS	1.72	1.06–2.77	0.027	1.50	0.89–2.50	0.125
Constant (baseline odds)	0.20			0.46		

**Note**: Adjusted for age, sex, income, physical activity, diet, cigarette, and shisha smoking, education.

#### Speed of Reaction

After multivariable logistic regression, MetS was associated with a 50% increase in the odds of poor speed of reaction (OR 1.5, 95% CI 0.89–2.50), but with weak evidence against the null hypothesis (P=0.125). Having 1–2 MetS components was also associated with higher odds of poor speed of reaction, but this was neither clinically nor statistically significant (OR 1.11, 95% CI 0.64 −1.80, P=0.677) (Table 3). In stratified analysis, in participants who did not attain tertiary level education, compared to having no MetS, MetS showed an almost 3-fold significantly higher odds of having poor speed of reaction score (OR 2.9, 95% CI 1.18–7.15, p = 0.021), while no association was observed in those with tertiary education (Supplementary Table 5). For MetS components, dysglycaemia and raised waist circumference were associated with higher odds of poor speed of reaction, while the other components did not show significant associations with poor speed of reaction (Supplementary Table 4).

## Discussion

In this cross-sectional analysis of middle-aged and elderly adults without cognitive dysfunction diseases in Qatar, we found that MetS was associated with poor cognitive function in the domains of psychomotor coordination and motor speed and short-term visual memory. In addition, we found that certain MetS components, such as dysglycaemia and raised waist circumference were associated with poor psychomotor coordination and motor speed. In stratified analysis, MetS was associated with poor cognitive function in those withouttertiary education, but not in those with tertiary education.

We found a strong association between MetS and poor short-term visual memory, with MetS increasing the odds of poor performance in the PAL memory test by 76%. Our findings were in line with other studies.^11^,^12^ The precise mechanisms by which MetS could influence cognitive functions remain unknown, but it has been hypothesised that there are neuroanatomical changes in individuals with MetS and that these are likely due to MetS-induced alterations in the hippocampal region of the brain, which plays an essential role in memory.^13^,^14^ It has also been suggested that increased inflammation and endothelial dysfunction in patients with MetS increase the risk for neurological degeneration.^15^ Among all MetS components, blood pressure was the most associated with increased odds of poor memory function in MetS patients with an OR of 1.4, followed by dysglycemia, HDL, and raised waist circumference. However, we observed no association with raised triglycerides. The association of hypertension with poor short term memory in individuals with MetS has been poorly studied, and research is scarce. However, previous explanatory models hypothesised that increased carotid artery stiffness is associated with cognitive impairment, specifically vascular dementia.^16^ For dysglycemia, a previous study performed on middle-aged patients identified that dysglycemia was clinically associated with less grey matter density and reduced glucose metabolism in the frontotemporal regions of the brain.^17^ Further, in line with our findings, a cohort study following 1147 patients for 7 years found no association between levels of triglycerides and cognitive dysfunction.^18^ However, in contrast to the findings of these studies, a recent study that involved participants from Tianjin, China, aged 45 years and older with poor income showed an association between high triglycerides and cognitive decline.^19^ The researchers of the Chinese study^19^ suggested that the differences between these various studies might be related to the nutritional and metabolic status of the different populations. Other studies that have used triglyceride-glucose index (TyG), an insulin resistance biomarker (calculated from triglyceride values and fasting serum glucose), reported that an increased TyG index is strongly associated with the risk of developing cognitive decline.^20^,^21^ Another study found that in participants with type 2 diabetes, the TyG index could be used to predict the risk of developing mild cognitive decline.^22^

In the present study, we found that participants with MetS had a 50% increase in odds of poor speed of reaction scores, although this change was not statistically significant . This finding is consistent with that of a previous study that reported an association between MetS and poor speed of reaction in the elderly.^23^ It has been suggested that speed of reaction is altered due to changes in fractional anisotropy, which is a measure of connectivity in the brain.^23^ We observed that the association between individual components of MetS and speed of reaction differed across the different risk factors. Participants with raised waist circumference had the highest odds of poor speed of reaction scores with an OR of 1.53, followed by dysglycemia with an OR of 1.38. To the best of our knowledge, there have been no studies that have investigated the association between waist circumference in MetS and cognition. However, a few previous studies that were conducted specifically on individuals with diabetes mellitus have found an association between waist circumference and cognitive impairment and dementia.^24^,^25^ One study showed that participants with obesity had damaged brain structure on neuroimaging, which included loss of brain volume and brain atrophy particularly of the grey matter, suggesting that inflammation related to obesity might cause cognitive decline.^26^ It should be noted that excess macronutrients of the adipose tissues stimulate the release of inflammatory mediators, such as tumor necrosis factor α and interleukin 6, and reduce the production of adiponectin, predisposing to a pro-inflammatory state and oxidative stress.^27^ The increased level of interleukin 6 stimulates the liver to synthesize and secrete C-reactive protein.^27^ As a risk factor, inflammation is an imbedded mechanism of developing cardiovascular diseases including coagulation, atherosclerosis, MetS, insulin resistance, and diabetes mellitus.^27^ Our current findings extend those from other studies of waist circumference by showing an association with MetS and distinguishing among cognitive domains, namely speed of reaction, which reflects connectivity in the brain. These findings indicate the importance of clinical screening for cognitive decline and early intervention in middle-aged patients with MetS to slow the progression of memory decline. Interventions and cognitive function screening and interventions in those at high risk of cognitive impairment should be added to the management of MetS.^28^

A narrative review concluded that dietary intervention and support of the gut-brain axis are potential early interventions to decrease the risk of developing cognitive dysfunction.^29^ It is noteworthy that interest in investigating the interaction between gut microbiome and neurological function is increasing. Specific microbial-derived metabolites present in individuals with MetS, such as trimethylamine N-oxide (TMAO), have been associated with cognitive decline.^30–32^ The precise underlying mechanism through which this occurs remains to be unidentified. However, animal studies have suggested that the metabolite leads to increased oxidative stress and neuroinflammation, and that the hippocampus area is affected.^33^,^34^ Given the well-established role of the hippocampus in memory formation,^35^ changes in its structure and/or function might contribute to the high increase in the odds of poor memory performance in the participants with MetS found in the present study. Additionally, education has been implicated to provide better resilience of cognition, thus, better cognitive reserve at higher levels of brain maintenance.^36^ Upon stratifying for education, we found that in participants with MetS who did not attain tertiary education, there was an almost 3-fold increase in odds of poor speed of reaction, and an odds of 2.51 for poor memory performance. In a longitudinal study conducted on an older population, it was found that in participants with higher education there was lesser rapid decline in cognition early in follow-up, however, with moderately faster decline in later follow-up.^37^

One limitation of our study is that the CANTAB tests done by the QBB only provided information about memory performance and speed of reaction, without testing other cognitive domains. The cross-sectional nature of this study implies that temporality cannot be assumed in this study. There was also lack of concurrent functional MRI of brain activity, which could identify any early cerebral abnormalities, especially in the middle-aged population.^38^ To our knowledge, this is also the first study to investigate the risk of developing cognitive decline in middle-aged individuals with MetS in the Middle East, and therefore adds a valuable contribution to this under-researched field. Future studies are needed to study the required length of exposure to MetS to develop cognitive dysfunction. In addition, further research can investigate if early administration of therapeutic agents would be effective in preventing cognitive decline.

## Conclusion

In middle-aged and elderly participants in the Middle East, MetS and some of its components were strongly associated with diminished short-term visual memory, psychomotor coordination and motor speed. Our findings suggest a need for screening and prevention of cognitive function in individuals with MetS, not only in the older populations, but also in the middle-aged.
